# CLDN6 inhibits breast cancer metastasis through WIP-dependent actin cytoskeleton-mediated autophagy

**DOI:** 10.1186/s13046-023-02644-x

**Published:** 2023-03-20

**Authors:** Yuan Dong, Qiu Jin, Minghao Sun, Da Qi, Huinan Qu, Xinqi Wang, Chengshi Quan

**Affiliations:** The Key Laboratory of Pathobiology, Ministry of Education, College of Basic Medical Sciences, Jilin University, 126 Xinmin Avenue, Changchun, 130021 Jilin China

**Keywords:** CLDN6, Breast cancer, Metastasis, Autophagy, WIP, Actin cytoskeleton

## Abstract

**Background:**

As a breast cancer suppressor gene, CLDN6 overexpression was found to inhibit breast cancer metastasis in our previous studies, but the specific mechanism remains unclear. This study aimed to clarify the role and mechanism of CLDN6 in inhibiting breast cancer metastasis.

**Methods:**

Western blot, immunofluorescence and transmission electron microscopy were performed to detect autophagy. Wound healing, transwell assays and lung metastasis mouse models were used to examine breast cancer metastasis. Phalloidin staining and immunofluorescent staining were used to observe actin cytoskeleton. mRNA seq, RT-PCR, western blot, chromatin immunoprecipitation, dual luciferase reporter assay, co-immunoprecipitation and immunofluorescence were performed to define the molecular mechanism. The expression levels and clinical implication of CLDN6, WIP and LC3 in breast cancer tissues were evaluated using immunohistochemistry.

**Results:**

We demonstrated that CLDN6 inhibited breast cancer metastasis through autophagy in vitro and vivo. We unraveled a novel mechanism that CLDN6 regulated autophagy via WIP-dependent actin cytoskeleton assembly. Through its PDZ-binding motif, overexpressed CLDN6 interacted with JNK and upregulated JNK/c-Jun pathway. C-Jun promoted WIP expression at the transcriptional level. Notably, we observed c-Jun transcriptionally upregulated CLDN6 expression, and there was a positive feedback loop between CLDN6 and JNK/c-Jun. Finally, we found that CLDN6, WIP and LC3 expression correlated with each other, and WIP expression was significantly associated with lymph node metastasis of breast cancer patients.

**Conclusions:**

The data provide a new insight into the inhibitory effects of CLDN6-mediated autophagy on breast cancer metastasis, and revealed the new mechanism of CLDN6 regulating autophagy through WIP-dependent actin cytoskeleton. Our findings enrich the theoretical basis for CLDN6 as a potential biomarker for breast cancer diagnosis and therapy.

**Supplementary Information:**

The online version contains supplementary material available at 10.1186/s13046-023-02644-x.

## Introduction

CLDNs are the main components of tight junctions of epithelial cells. CLDN6 is a member of the CLDNs family, containing four transmembrane helices and a COOH-terminal PDZ-binding motif (PBM) [1]. In addition to traditional functions of CLDN6 in permeability regulation and barrier formation [2–5], CLDN6 connects proteins containing PDZ domain or PBM through its PBM, regulating intracellular signaling pathways to affect the malignant phenotype of cancer [6–11]. Previously, we have cloned CLDN6 as a candidate suppressor gene of breast cancer from COP rat mammary epithelial cells for the first time [12]. We proved that CLDN6 was low expressed in breast cancer and CLDN6 overexpression suppressed breast cancer metastasis [13], but the underlying mechanism is not well understood.

Autophagy is a lysosome-dependent catabolic process induced by various cellular stress conditions [14]. As a mechanism of cell survival, autophagy has recently been recognized as one of the key mechanisms of cancer metastasis. Autophagy plays two distinct roles in cancer metastasis: promotion and inhibition, depending on cancer cell type, the tumor microenvironment and the stage of tumor progression [14, 15]. Our group revealed that ERβ inhibited breast cancer cells migration and invasion through CLDN6-mediated autophagy [6]. However, the role and relevant mechanism of CLDN6-mediated autophagy in regulating breast cancer metastasis needs to be explored.

Recent studies have suggested that actin cytoskeleton plays a critical role in autophagy by acting on the vesicle membrane. During autophagosome formation, actin cytoskeleton assembles inside and outside the autophagosome to generate power or provide scaffolds, stabilizing and promoting membrane bending [16, 17]. Actin polymerization generates branched actin networks on the vesicle surface to drive the fusion of mature autophagosome with lysosomes [18–20], and promotes autolysosome reformation [21]. In previous mRNA microarray, we observed that differential genes enriched in regulation of actin cytoskeleton in CLDN6-overexpressing breast cancer cells [7], which indicated that CLDN6 may mediate autophagy by affecting actin cytoskeleton. Nevertheless, the mechanism by which CLDN6 affects actin cytoskeleton assembly on autophagosome remains unclear.

Nucleation-promoting factors (NPFs), is a class of proteins that activates the Arp2/3 complex to promote actin cytoskeleton assembly [22]. It has been reported that NPFs and Arp2/3 complex are recruited to autophagosome and promoted actin cytoskeleton assembly on the membrane [18–21, 23, 24]. NPFs contains four families: WAVE, WASP, WASH, and WHAMM families [25]. WASP-interacting protein (WIP), the WASP binding protein, mediates actin cytoskeleton assembly by activating the Arp2/3 or binding actin filaments [26]. However, the role of WIP in autophagy has not been studied. Using mRNA sequencing we found WIP expression was upregulated in CLDN6-overexpressing breast cancer cells. Thus, we speculated that CLDN6 may regulate WIP to affect actin cytoskeleton during autophagy, and further inhibit breast cancer metastasis.

In present study, we reported that CLDN6 regulated breast cancer metastasis via autophagy in vitro and vivo. CLDN6-mediated autophagy was dependent on regulation of WIP in actin cytoskeleton assembly. We further illustrated the molecular mechanism of CLDN6 regulating WIP at the transcriptional level through JNK/c-Jun pathway, and unexpectedly discovered the feedback regulation between JNK/c-Jun and CLDN6. Finally, we investigated the correlation between CLDN6, WIP and LC3 expression in breast cancer tissues and the relationship between them and clinicopathological characteristics of breast cancer patients. In conclusion, our results clarified the role of CLDN6 in the relationship between cell homeostasis and cancer metastasis, and revealed the molecular mechanisms.

## Materials and methods

### Cell culture

Human breast cancer cell lines MCF-7 and MDA-MB-231 were cultured in Dulbecco’s modified Eagle’s medium (DMEM; Meilune, Dalian, China) containing 10% fetal bovine serum (FBS; Gibco, Carlsbad, CA, USA) at 37 °C in a humidified atmosphere with 5% CO_2_.

### Western blot

Western blot assay was performed as previously described [7]. Antibodies used in this study were listed in Supplementary materials.

### Immunofluorescent staining (IF)

The IF assay was performed as previously described [8]. Antibodies used in IF were listed in Supplementary materials.

### Wound healing assay

The cells were cultured in 12-well culture plates, and were scratched using 200 μl pipette tips. Then cells were cultured in medium without FBS. Images were taken at 0 h and 48 h to define the width of the wounded area.

### Transwell migration and invasion assays

The migration and invasion assays were performed using Transwell chamber (Corning, Lowell, MA, USA). For migration, cells were placed in the upper chamber with serum-free medium (2.5 × 10^4^ cells), and the bottom of the chamber contained the DMEM medium with 10% FBS. For invasion, the chamber was covered with Matrigel (Corning, Lowell, MA, USA), and the subsequent steps were similar to migration assay. After 24 h incubation, cells were fixed in 4% paraformaldehyde and stained with 0.1% crystal violet. The number of migrated or invaded cells was counted in three random fields at × 200 magnification.

### Phalloidin staining

Cells were fixed with 4% paraformaldehyde for 10 min, and permeabilized with 0.1% TritonX-100 for 20 min. Actin cytoskeleton was stained using TRITC-Phalloidin (Abcam, Cambridge, UK) at room temperature for 1 h. Then cells were stained with DAPI for 5 min. Images were collected with fluorescence microscopy (Olympus, Japan).

### RT-PCR

Total RNA was extracted from cells using the TRIzol reagent (Invitrogen, Carlsbad, CA, USA), and was converted to cDNA using MonaScript RT All–in–One Mix (Monad, Wuhan, China). RT-PCR was performed with 2 × TransTaq®-T PCR SuperMix (Transgen, Beijing, China). The primers were listed in Supplementary materials.

### Transfection

The specific short hairpin RNA (shRNA) for WIP, c-Jun and JNK, with negative control shRNA were purchased from PPL Genebio Technology (Nanjing, China). The transfection process was performed as previously described [9].

### Transmission electron microscopy (TEM)

Samples were prepared as previously described [6]. Images were taken with transmission electron microscopy (FEI Tecnai Spirit, USA).

### Chromatin immunoprecipitation (ChIP) assay

ChIP assay was performed according to manufacturer’s protocol (Beyotime, Shanghai, China). DNA was extracted by DNA Purification Kit (Beyotime, Shanghai, China) and eluted for PCR. Primer sequences were listed in Supplementary materials.

### Dual luciferase reporter assay

Cells seeded into 12-well dishes were transfected with Wild-type (WT) and mutant (Mut) reporter plasmids expressing firefly luciferase and renilla luciferase reporter plasmid (pRL-TK). WT plasmids contained WIP promoter fragment (− 2000/ + 100 bp), and Mut plasmids altered the base arrangement of the site where c-Jun bound to WIP gene promoter. After transfection 48 h, cells were collected using Double-Luciferase Reporter Assay Kit (Transgen, Bei jing, China) and analyzed by the dual-luciferase reporter gene assay system (Promega, Madison, WI, USA). The ratio of firefly luciferase activity to Renilla luciferase activity was determined. Reporter plasmids were purchased from PPL Genebio Technology (Nanjing, China).

### Co-immunoprecipitation (Co-IP) assay

The Co-IP assay was performed as previously described [7]. Antibodies used were listed in Supplementary materials.

### Animal experiments

All animal experiments were approved by the Animal Care and by the Experimental Animal Ethical Committee of Jilin University. The MDA-MB-231/Vector, MDA-MB-231/CLDN6, and MDA-MB-231/CLDN6-shWIP cells (1 × 10^7^ cells/ml, 100 μl/ mouse) were injected into BALB/c-nu female nude mice (4–5 weeks old) via the tail vein to establish a tumor lung metastasis model. In the Chloroquine (CQ)-treated mice model, MDA-MB-231/Vector and MDA-MB-231/CLDN6 cells were injected. After one week, the animals were intravenously administered CQ (30 mg/kg body weight) every three days. After three weeks, D-luciferin at a dose of 1.5 mg/10 g body weight was injected intraperitoneally into mice, and 10 min later, luciferase imaging (Xenogen IVIS-200) was performed. Then the mice were euthanized, and the lungs of mice were removed and fixed in 10% neutral formalin. The metastasis ability was detected by hematoxylin and eosin (H&E) staining.

### Immunohistochemistry (IHC)

Human breast cancer tissue microarray (TMA; HBreD070CS02) was purchased from Shanghai Outdo Biotech Company (Shanghai, China). The IHC assay was performed according to manufacturer’s protocol (KIT–9710, MXB Biotechnology, Fuzhou, China). IHC scores are a combination of positive intensity (0, none; 1, weak; 2, moderate; 3, strong) and a proportion of positive cells (≤ 25%: 1; 26–50%: 2; 51–75%: 3; ≥ 75%: 4). Antibodies used were listed in Supplementary materials.

### Statistical analysis

All statistical analyses were performed using GraphPad Prism 8.0 (GraphPad, San Diego, CA, USA). The data were presented as the mean ± standard deviation (SD) of at least three independent experiments. Two independent samples with a normal distribution were compared by Two-sided Student’s t-test when the variance was homogeneous, and by Welch’s t-test when the variance is not homogeneous. A nonparametric test was used to assess data with an abnormal distribution. The relationships between clinical parameters and indicates gene expression were determined by using two-sided Chi-square test. Correlations between proteins were analyzed using Pearson’s correlation coefficients. *P* < 0.05 was considered statistically significant.

## Results

### CLDN6 regulates breast cancer metastasis via autophagy in vitro and vivo

**Fig. 1:**
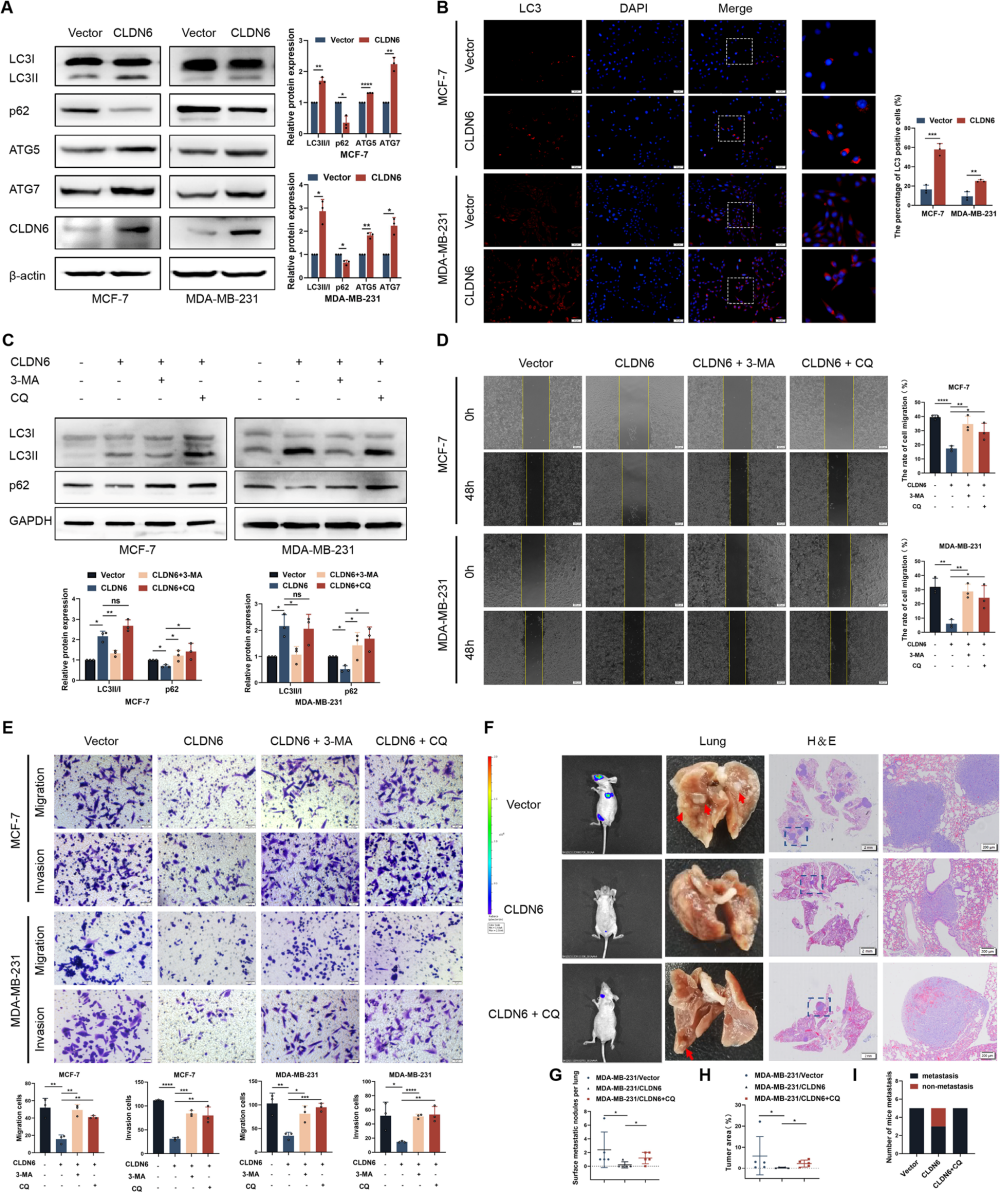
CLDN6 regulates breast cancer metastasis via autophagy in vitro and vivo. **A** Western blot showed levels of autophagy-related proteins in MCF-7 and MDA-MB-231 cells with or without CLDN6 overexpression. **B** LC3 puncta was visualized by IF analysis, and the percentage of LC3 positive cells was quantified, zoomed-in image (right). Scale bar: 50 μm. **C** Western blot showed levels of autophagy after treated with 2 mM 3-MA or 25 μM CQ for 24 h. (**D-E**) Wound healing assay and transwell migration and invasion assays were performed in CLDN6-overexpression cells treated with 3-MA or CQ. Scale bar: 200 μm (**D**) and 50 μm (**E**). Results of (**A − E**) were from three independent experiments. **F** Bioluminescence imaging (BLI) was used to monitor metastases of BALB/c-nu mice. Representative lung metastatic nodes (red arrowheads). Representative H&E staining of lung sections from the three groups (*n* = 5 mice/group), Scale bar: 2 mm (left), 200 μm (right). **G** The number of surface metastatic nodes per lung in each group. **H** The areas were quantified with Image-Pro Plus and the tumor ratio (%) was determined as (tumor area)/(total lung area) × 100%. **I** The number of mice with lung metastasis was counted in each group. *P < 0.05, **P < 0.01, ***P < 0.001, ****P < 0.0001 and ns: no significance

Previously, we have proved that CLDN6 expression is decreased in human breast cancer compared with that in normal breast tissues. Overexpression of CLDN6 inhibits the metastasis of breast cancer, but the underlying mechanism is incompletely understood. Since the importance of autophagy in tumor metastasis, we examined the effect of CLDN6 in regulating autophagy in breast cancer cells. Using lentivirus transduction, we constructed breast cancer MCF-7 and MDA-MB-231 cell lines with stable overexpression of CLDN6 (Supplementary Fig. 1A, B). Western blot analysis revealed that CLDN6 overexpressed increased levels of LC3II/I, ATG5, and ATG7 (Fig. 1A). Consistent with this, we observed that CLDN6 increased the percentage of cells containing LC3 positive puncta using IF analysis (Fig. 1B). These data indicated an increase in the synthesis of autophagosome. Since decreased p62 level was associated with increased autophagic flux [27], CLDN6 overexpression decreased p62 protein level (Fig. 1A), indicating enhanced autophagic flux. Together, our results suggested that overexpressed CLDN6 induced autophagy and enhanced autophagic flux in breast cancer cells.

We further investigated whether CLDN6 inhibited the migration and invasion of breast cancer cells through autophagy, we used 3-Methyladenine (3-MA) to inhibit the formation of autophagosome in the early stage of autophagy, and CQ to inhibit the fusion of autophagosome and lysosomes in the late stage of autophagy, respectively (Fig. 1C). Wound healing and transwell migration and invasion assay revealed that CLDN6 overexpression in MCF-7 and MDA-MB-231 cells decreased cell migration and invasion, while 3-MA and CQ rescued cell migration and invasion (Fig. 1D, E). These data indicated that CLDN6 inhibited breast cancer cell migration and invasion via autophagy. In addition, inhibition of autophagy rescued the inhibition of N-cadherin and vimentin induced by CLDN6 overexpression (Supplementary Fig. 2), indicating that CLDN6-mediated autophagy inhibited cell migration and invasion by suppressing EMT.

To further validate the role of CLDN6-induced autophagy in breast cancer metastasis in vivo, we established lung metastasis mouse models with BALB/c-nu mice. Considering that CQ as an autophagy inhibitor is being investigated in many clinical trials [28], we used CQ to inhibit autophagy in vivo experiments. The following three experimental groups were tested: MDA-MB-231/NC (control MDA-MB-231 cells), MDA-MB-231/CLDN6 (CLDN6-overexpressing MDA-MB-231 cells), and MDA-MB-231/CLDN6 + CQ (CLDN6-overexpressing MDA-MB-231 cells treated with CQ). Tumor growth was examined by luciferase imaging, and lung and tissue sections were analyzed by H&E staining. Mice injected with MDA-MB-231/CLDN6 exhibited decreased occurrence of lung metastasis compared with NC, while this phenomenon was rescued by treatment with CQ (Fig. 1F-I). These results indicated that CLDN6 regulated breast cancer metastasis via autophagy in vivo.

### CLDN6 mediates autophagy by regulating actin cytoskeleton

**Fig. 2:**
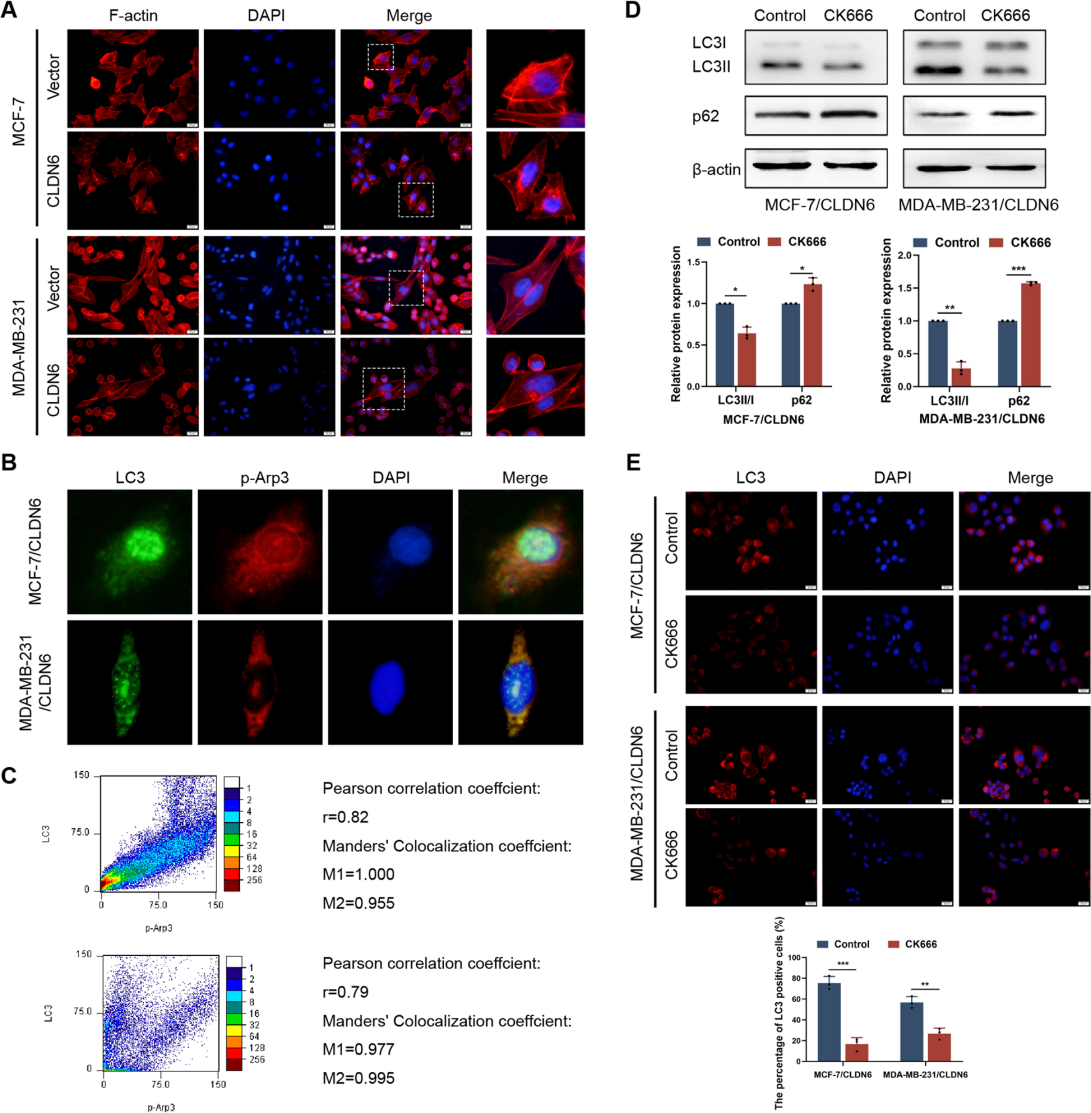
CLDN6 mediates autophagy by regulating actin cytoskeleton. **A** F-actin labeled with Rhodamine phalloidin in MCF-7 and MDA-MB-231 cells with or without CLDN6 overexpression, zoomed-in image (right). Scale bar: 20 μm. **B** IF analysis was performed to detect the colocalization of LC3 (green) with p-Arp3 (red) in MCF-7/CLDN6 and MDA-MB-231/CLDN6 cells. Scale bar: 20 μm. **C** ImageJ was used for colocation analysis of p-Arp3 and LC3. Pearson correlation analysis showed that r > 0.5. Manders^,^ colocation coefficient showed that M1 > 0.5 and M2 > 0.5. **D** The expression of LC3 and p62 was detected by western blot in MCF-7/CLDN6 and MDA-MB-231/CLDN6 cells treated with or without 150 μM CK666 for 4 h. **E** IF analysis showed that the percentage of LC3 positive cells was decreased after CK666 treatment. All results were from three independent experiments. Scale bar: 20 μm. *P < 0.05, **P < 0.01 and ***P < 0.001

Currently, the regulatory mechanism of CLDN6-mediated autophagy is unclear. Recent studies have revealed that actin cytoskeleton plays a critical role in autophagy [16]. Using mRNA microarray, we previously observed that differential genes enriched in the regulation of actin cytoskeleton in CLDN6-overexpressing breast cancer cells [7]. To further investigate, we used phalloidin staining to observe the structure and distribution of F-actin. We observed abundant parallel F-actin fibers throughout the cytoplasm in control cells. However, in CLDN6-overexpressing cells, parallel F-actin fibers disappeared and lots of punctuated F-actin diffused around the nucleus (Fig. 2A). Our observations supported that CLDN6 promoted rearrangement of actin cytoskeleton.

Next, we investigated whether CLDN6 mediated autophagy by regulating actin cytoskeleton. Arp3, a subunit of the Arp2/3 complex, acts as a marker of the branched actin network. We found that p-Arp3 was co-localized with LC3 (Fig. 2B, C), which indicated that actin cytoskeleton was localized in autophagosome during CLDN6-mediated autophagy. Furthermor, we treated CLDN6-overexpressing breast cancer cells with CK666, a cell-permeable drug that inhibits Arp2/3 activity, to inhibit actin cytoskeleton formation. Our results showed that CK666 reduced levels of LC3II and increased p62 expression (Fig. 2D). Moreover, CK666 decreased the percentage of cells containing LC3 positive puncta (Fig. 2E). These data suggested that CLDN6-mediated autophagy was dependent on actin cytoskeleton assembly.

### CLDN6 increases WIP expression to regulate actin cytoskeleton and mediate autophagy

**Fig. 3:**
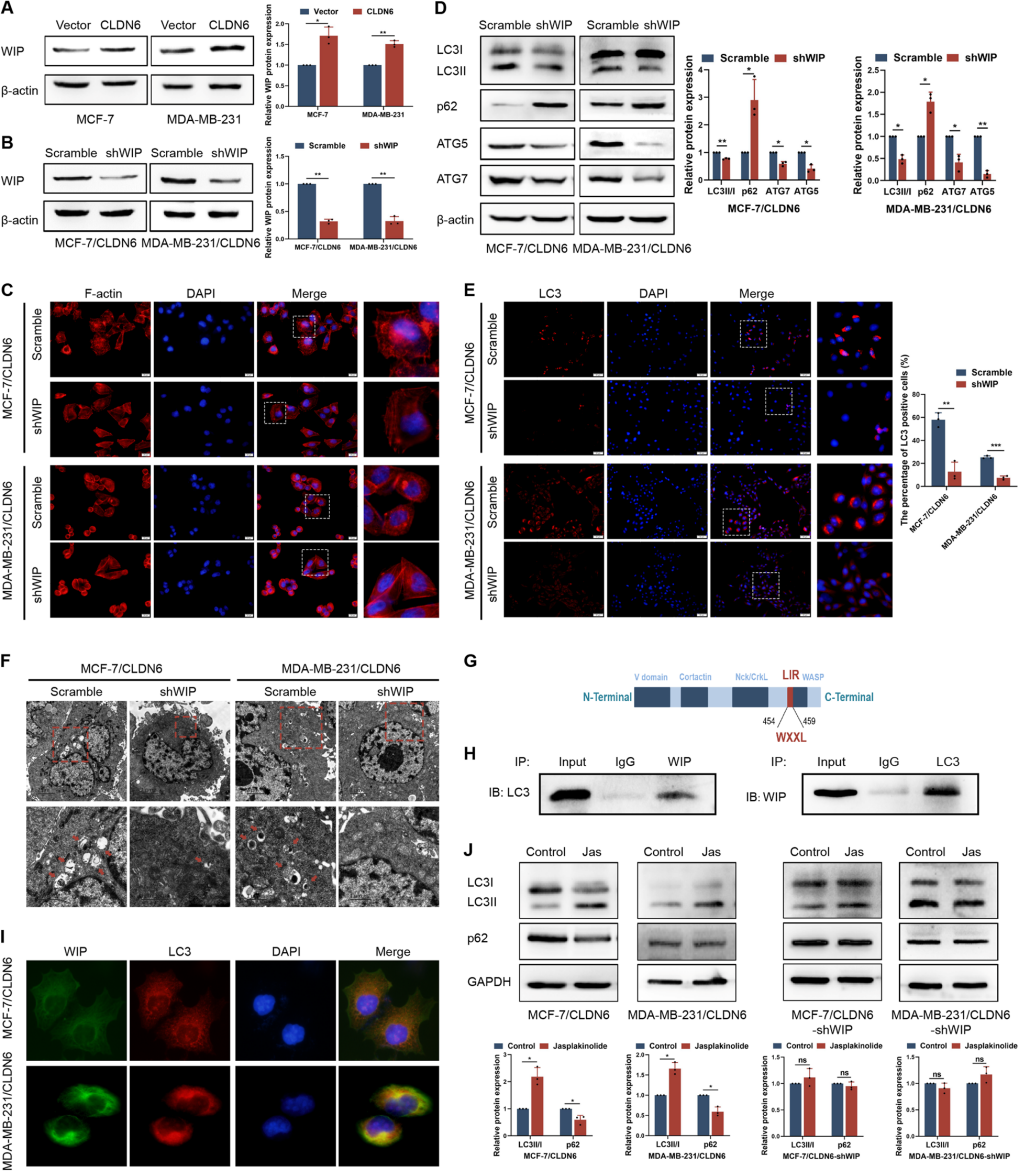
CLDN6 increases WIP expression to regulate actin cytoskeleton-mediated autophagy. **A** Western blot showed levels of WIP protein in MCF-7 and MDA-MB-231 cells with or without CLDN6 overexpression. **B** Western blot showed levels of WIP protein in MCF-7/CLDN6 and MDA-MB-231/CLDN6 cells with or without WIP knockdown. **C** F-actin labeled with Rhodamine phalloidin in MCF-7/CLDN6 and MDA-MB-231/CLDN6 cells with or without WIP knockdown, zoomed-in image (right). Scale bar: 20 μm. **D** Western blot showed levels of autophagy-related proteins in MCF-7/CLDN6 and MDA-MB-231/CLDN6 cells with or without WIP knockdown. Results were from three independent experiments. **E** IF analysis showed LC3 positive cells in MCF-7/CLDN6 and MDA-MB-231/CLDN6 cells with or without WIP knockdown, zoomed-in image (right). Scale bar: 50 μm. **F** TEM analysis was performed on MCF-7/CLDN6 and MDA-MB-231/CLDN6 cells with or without WIP knockdown. Control group displayed several autophagic vacuoles (red arrowheads) which were not observed in WIP knockdown cells. Scale bar: 2 μm (top) and 1 μm (bottom). **G** The potential LIR motifs of the WIP. **H** The interaction of WIP and LC3 was detected by Co–IP assay in MDA-MB-231/CLDN6 cells. **I** IF analysis was performed to detect the colocalization of WIP (green) with LC3 (red) in MCF-7/CLDN6 and MDA-MB-231/CLDN6 cells. Scale bar: 20 μm. **J** Western blot showed the expression of LC3 and p62 in MCF-7/CLDN6, MDA-MB-231/CLDN6, MCF-7/CLDN6-shWIP and MDA-MB-231/CLDN6-shWIP cells treated with or without 100 nM Jasplakinolide for 2 h. Results were from three independent experiments. *P < 0.05, **P < 0.01, ***P < 0.001 and ns: no significance

To investigate how CLDN6 regulated actin cytoskeleton during autophagy, we performed a mRNA sequencing using MCF-7/Vector and MCF-7/CLDN6. Comparing the differential genes with NPFs-related genes, we found WIP expression was upregulated (Supplementary Fig. 3A, B). RT-PCR and Western blot confirmed that CLDN6 overexpression increased the mRNA and protein expression of WIP in MCF-7 and MDA-MB-231 cells (Supplementary Fig. 3C; Fig. 3A). IHC of lung metastatic tumor tissues of mice showed the expression intensity of CLDN6 was consistent with WIP and LC3 (Supplementary Fig. 3D). Next, we knocked down WIP in MCF–7/CLDN6 and MDA–MB–231/CLDN6 cells. We confirmed that shRNA targeting WIP noticeably reduced WIP expression in MCF-7 and MDA-MB-231 cells compared with the negative control shRNA (Fig. 3B).

To test the effect of CLDN6 on actin polymerization through WIP, we used phalloidin staining. The results showed that WIP knockdown inhibited F-actin aggregated around the nucleus in CLDN6-overexpressing breast cancer cells (Fig. 3C), which suggested that CLDN6 regulated actin cytoskeleton through WIP.

We then evaluated whether WIP was critical for CLDN6-mediated autophagy in breast cancer cells. We observed that WIP knockdown decreased levels of LC3II/I, ATG5, and ATG7 in CLDN6-overexpressing cells (Fig. 3D). The percentage of cells containing LC3 positive puncta was downregulated following WIP knockdown (Fig. 3E). Consistently, TEM results showed that WIP knockdown decreased the number of autophagosome (Fig. 3F). Furthermore, elevated p62 levels indicated the suppression of autophagic flux after WIP knockdown (Fig. 3D). Together, these data suggested that CLDN6 mediated autophagy by upregulating WIP expression.

The LIR motif consists of a conserved W/F/YxxL/I/V sequence, and proteins containing this motif can directly interact with LC3 protein [29]. Using the iLIR web server (https://ilir.warwick.ac.uk/) [30], we found that WIP contained a LIR motif (Fig. 3G). Consistent with this finding, Co-IP experiment revealed that WIP and LC3 interacted with each other in MDA-MB-231/CLDN6 cells (Fig. 3H). Moreover, we observed that WIP colocalized with LC3 in CLDN6-overexpressing breast cancer cells (Fig. 3I; Supplementary Fig. 4A, B). Thus, in the presence of CLDN6 overexpression, WIP may be recruited to autophagosome by LC3 to regulate autophagy.

Next, we studied whether WIP regulated autophagy through actin cytoskeleton. We treated CLDN6-overexpressing breast cancer cells with Jasplakinolide to prevent F-actin depolymerization, and found that Jasplakinolide treatment promoted autophagy. However, Jasplakinolide lost the effect after WIP knockdown (Fig. 3J). The results suggested that CLDN6 relied on WIP-mediated actin cytoskeleton assembly to promote autophagy.

### CLDN6 inhibits breast cancer metastasis via the upregulation of WIP expression in vitro and vivo

**Fig. 4:**
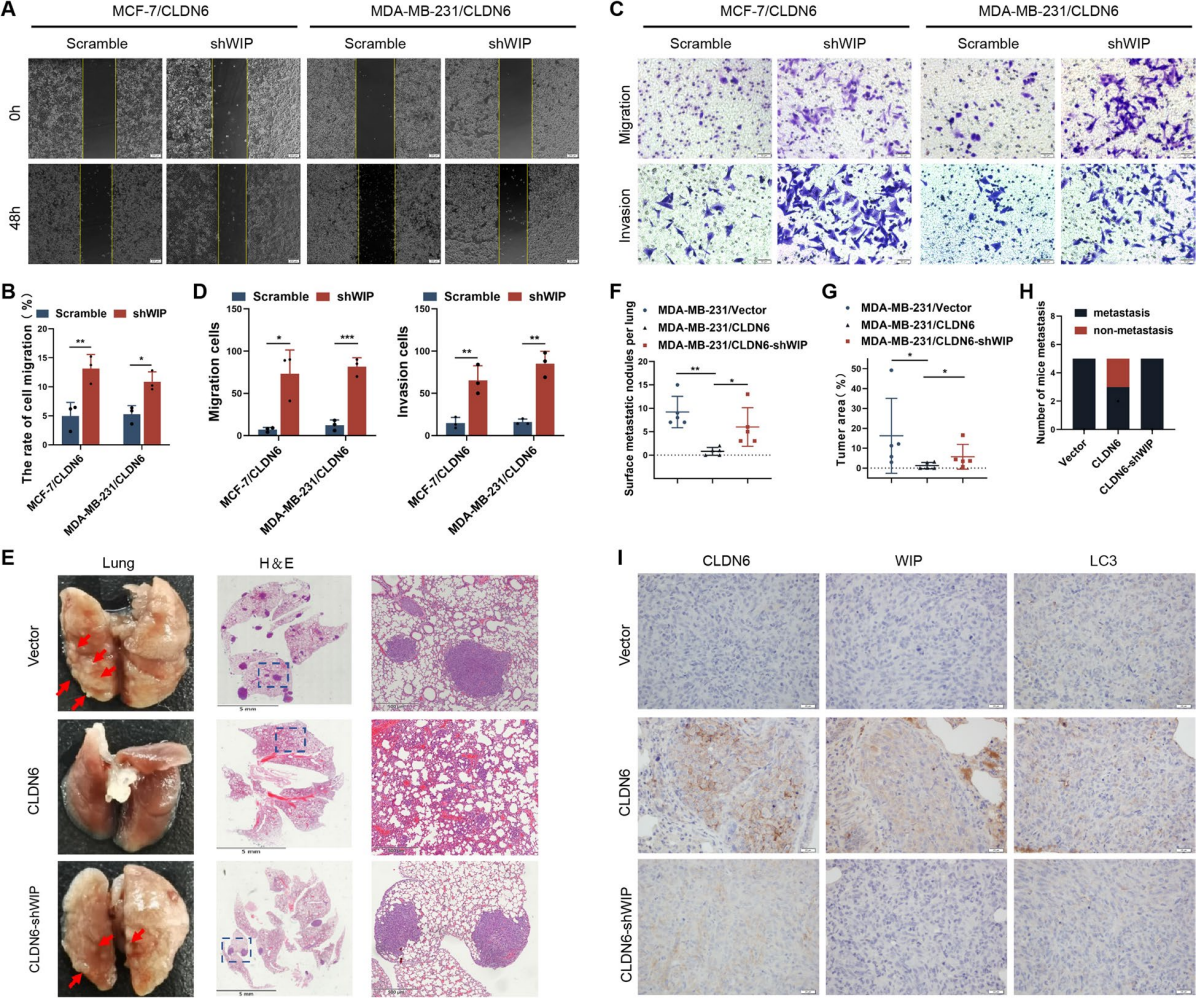
CLDN6 inhibits breast cancer metastasis via the upregulation of WIP expression in vitro and vivo. **A-D** Wound healing assay and transwell migration and invasion assays were performed in MCF-7/CLDN6 and MDA-MB-231/CLDN6 cells with or without WIP knockdown. Results were from three independent experiments. Scale bar: 200 μm (**A**) and 50 μm (**C**). **E** Representative examples of lungs with metastatic foci (red arrowheads) are shown. Representative H&E staining of lung sections from the three groups (*n* = 5 mice/group), zoomed-in image (right). Scale bar: 5 mm (left), 500 μm (right). **F** The number of surface metastatic nodes per lung in each group. **G** The areas were quantified with Image-Pro Plus and the tumor ratio (%) was determined as (tumor area)/(total lung area) × 100%. **H** The number of mice with lung metastasis was counted in each group. **I** Representative IHC images of CLDN6, WIP, and LC3 in the lung metastatic tumor tissues. Scale bar: 20 μm. *P < 0.05, **P < 0.01 and ***P < 0.001

The critical role of WIP in CLDN6-mediated autophagy led us to hypothesize that CLDN6 may inhibit breast cancer metastasis by upregulating WIP expression. To test this hypothesis, we performed studies in vitro and vivo. Wound healing and transwell migration and invasion assay revealed that WIP knockdown increased cell migration and invasion in CLDN6-overexpression breast cancer cells (Fig. 4A-D), which indicated that CLDN6 inhibited migration and invasion of breast cancer cells through WIP. Next, we established lung metastasis mouse models using MDA-MB-231/NC cells, MDA-MB-231/CLDN6 cells and MDA-MB-231/CLDN6-shWIP cells (CLDN6-overexpressing and WIP-knockdown MDA-MB-231 cells). Lung metastasis was determined by macroscopic observations and H&E staining. The results showed that CLDN6 reduced the number of lung surface metastatic nodules and the area of lung metastasis, while WIP knockdown reversed this inhibitory effect of CLDN6 (Fig. 4E-H). In addition, the protein expression of CLDN6, WIP and LC3 in the lung metastatic tumor tissues was tested by IHC. The group with MDA-MB-231/CLDN6 cells showed positive expression of CLDN6, WIP and LC3, whereas the expression of WIP and LC3 was negative after WIP knockdown (Fig. 4I). In summary, our results showed that CLDN6 inhibited breast cancer metastasis by upregulating WIP expression in vitro and vivo.

### CLDN6 upregulates WIP expression at transcriptional level via c-Jun

**Fig. 5:**
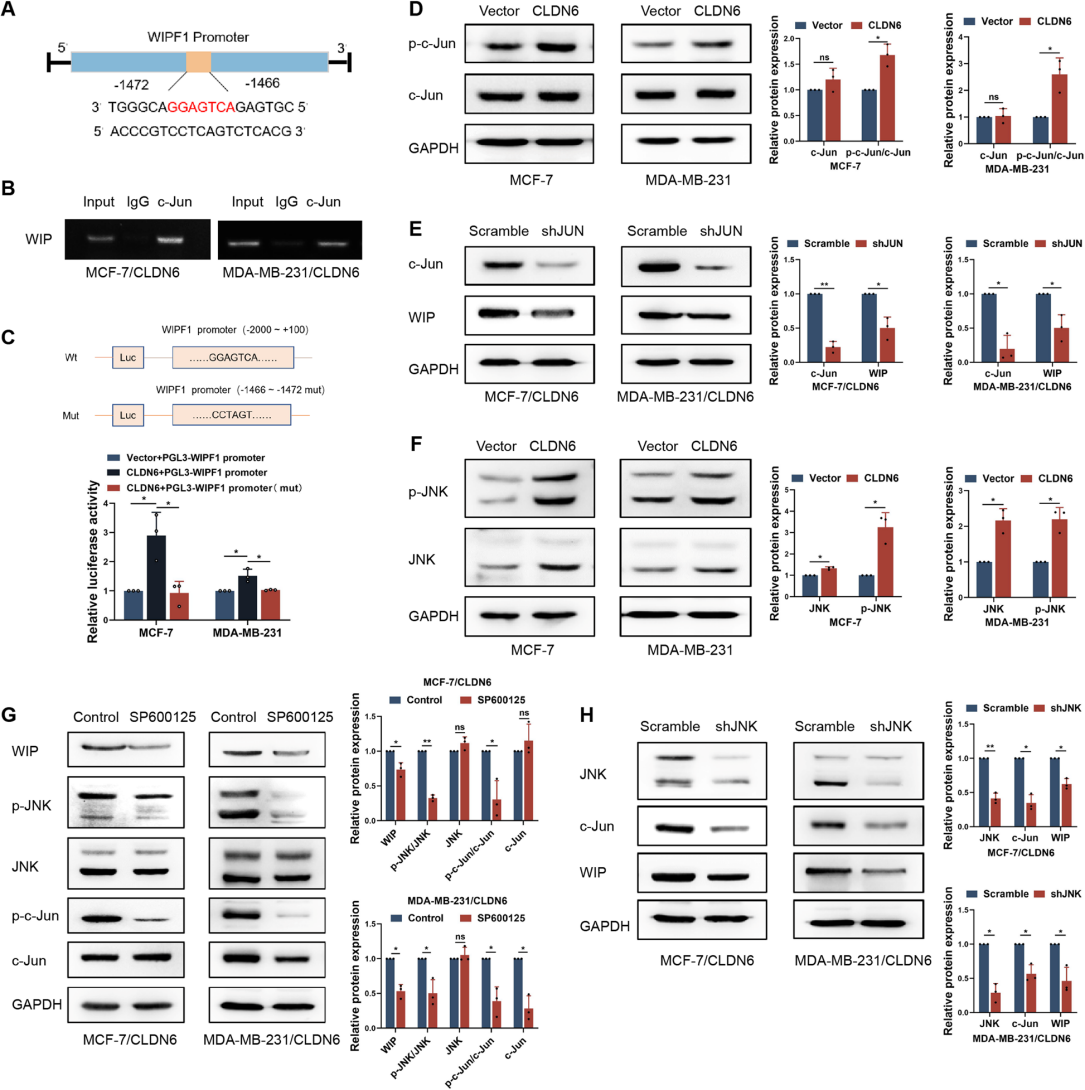
CLDN6 upregulates WIP at transcriptional level via JNK/c-Jun pathway.** A** The potential transcriptional binding site of c-Jun on the WIP promoter. **B** ChIP experiment determined the combination of c-Jun with the predicted site in the WIP promoter. **C** Cells were transfected with Wt or Mut WIP promoter reporter plasmid. At 48 h after transfection, promoter activity was analyzed using dual-luciferase assay. **D** Western blot showed levels of total and phosphorylated c-Jun in MCF-7 and MDA-MB-231 cells with or without CLDN6 overexpression. **E** Western blot showed levels of WIP protein in MCF-7/CLDN6 and MDA-MB-231/CLDN6 cells with or without c-Jun knockdown. **F** Western blot showed levels of total and phosphorylated JNK in MCF-7 and MDA-MB-231 cells with or without CLDN6 overexpression. **G** Western blot showed levels of WIP and JNK/c-Jun in MCF-7/CLDN6 and MDA-MB-231/CLDN6 cells treated with or without 15 μM SP600125 for 4 h. **H** Western blot showed levels of WIP and JNK/c-Jun in MCF-7/CLDN6 and MDA-MB-231/CLDN6 cells with or without JNK knockdown. Results were from three independent experiments. *P < 0.05, **P < 0.01, and ns: no significance

Next, we explored the specific mechanism of CLDN6 regulating WIP. Since CLDN6 upregulated WIP expression at the transcriptional level, we speculated that CLDN6 may influence transcription factors of WIP. Using the GCBI (https://www.gcbi.com.cn/) and JASPER website (https://jaspar.genereg.net/), we predicted that the transcription factor c-Jun can bind to the promoter region of WIP (Fig. 5A). Data from the TCGA demonstrated that WIP expression was positively correlated with c-Jun expression (Supplementary Fig. 5A). ChIP assay showed that c-Jun bound to the predicted site of WIP gene promoter in CLDN6-overexpressing breast cancer cells (Fig. 5B). Luciferase reporter gene assay showed that CLDN6 overexpression activated the WIP promoter activity (Fig. 5C). Moreover, we constructed a mutant luciferase reporter plasmids. Mutant plasmids altered the site where c-Jun bound to WIP gene promoter. We observed that the luciferase activity in CLDN6-overexpressing cells transfected with mutant plasmids was significantly reduced (Fig. 5C). The transcriptional activity of c-Jun is regulated by Ser63/Ser73 phosphorylation [31]. We also found that c-Jun phosphorylation was upregulated in CLDN6-overexpressing breast cancer cells (Fig. 5D), and c-Jun knockdown eliminated the promotion of WIP induced by CLDN6 overexpression (Fig. 5E; Supplementary Fig. 6A). These data suggested that CLDN6 upregulated WIP expression by increasing c-Jun transcriptional activity.

### JNK is required for c-Jun activity in CLDN6-overexpressing breast cancer cells

JNK leads to increased S63/73 phosphorylation and transcriptional activity of c-Jun [32]. According to the TCGA, WIP expression was positively correlated with JNK expression (Supplementary Fig. 5B). Our results showed CLDN6 overexpression enhanced JNK protein expression and phosphorylation level (Fig. 5F). Thus, we hypothesized that CLDN6 regulated WIP through JNK/c-Jun pathway. To validate this, we treated CLDN6-overexpressing breast cancer cells with the JNK inhibitor SP600125 [33]. Inhibition of JNK phosphorylation downregulated c-Jun phosphorylation and WIP expression (Fig. 5G). Consistent with this, JNK knockdown resulted in decreased expression of c-Jun and WIP (Fig. 5H). These results demonstrated that CLDN6 upregulated WIP expression at the transcriptional level through JNK/c-Jun pathway.

### CLDN6 interactes with JNK via the PDZ-binding motif

**Fig. 6:**
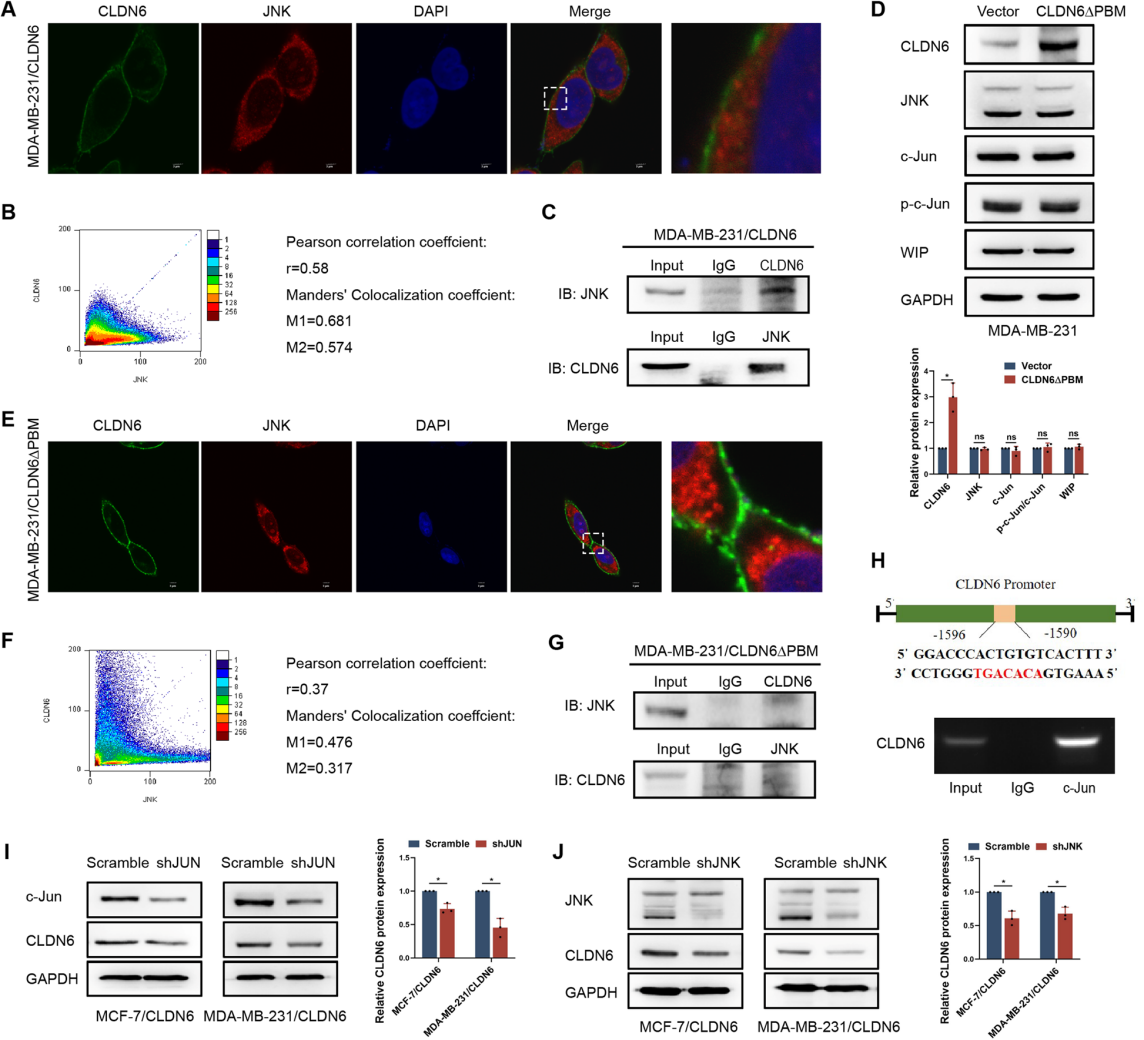
CLDN6 interacts with JNK via the PDZ-binding motif.** A** IF analysis was performed to detect the co-localization of CLDN6 (green) with JNK (red) in MDA-MB-231/CLDN6 cells, zoomed-in image (right). Scale bar: 3 μm. **B** ImageJ was used for colocation analysis. Pearson correlation analysis showed that r > 0.5. Manders, colocation coefficient showed that M1 > 0.5 and M2 > 0.5. **C** The interaction of CLDN6 and JNK was detected by Co–IP assay in MDA-MB-231/CLDN6 cells. **D** Western blot showed levels of JNK/c-Jun pathway and WIP in MDA-MB-231 cells with or without CLDN6ΔPBM overexpression. Results were from three independent experiments. **E** The interaction of CLDN6 and JNK was detected by Co–IP assay in MDA-MB-231/CLDN6ΔPBM cells. **F** IF analysis was performed to detect the co-localization of CLDN6 (green) with JNK (red) in MDA-MB-231/CLDN6ΔPBM cells. Scale bar: 3 μm. **G** ImageJ was used for colocation analysis. Pearson correlation analysis showed that r < 0.5. Manders^,^ colocation coefficient showed that M1 < 0.5 and M2 < 0.5. **H** The potential transcriptional binding site of c-Jun on the CLDN6 promoter, and was verified by ChIP assays. **I-J** JNK/c-Jun feedback regulates CLDN6 expression. **I** Western blot showed levels of CLDN6 protein in MCF-7/CLDN6 and MDA-MB-231/CLDN6 cells with or without c-Jun knockdown. **J** Western blot showed levels of CLDN6 protein in MCF-7/CLDN6 and MDA-MB-231/CLDN6 cells with or without JNK knockdown. Results were from three independent experiments. *P < 0.05 and ns: no significance

In terms of the mechanism by which CLDN6 affected JNK, we next observed the cellular localization of CLDN6 and JNK in MDA-MB-231 cells with CLDN6 overexpression by IF assay. CLDN6 was mainly localized in the cell membrane, and JNK co-localized with CLDN6 at the cell edge (Fig. 6A, B). The Co-IP results further validated the association between CLDN6 and JNK (Fig. 6C). We next investigated whether the interaction of CLDN6 with JNK was PBM-dependent. We created a mutant of CLDN6 (CLDN6ΔPBM) affecting its PBM. As shown in Fig. 6D, CLDN6ΔPBM failed to upregulate WIP and JNK protein expression as well as c-Jun activation. Moreover, the mutant CLDN6 was not able to interact with JNK (Fig. 6E-G). Taken together, these results demonstrated that CLDN6 interacted with JNK via its PBM, and then promoted the activation of JNK/c-Jun signaling.

### JNK mediated c-Jun activation feedback regulates CLDN6 expression

Several feedback mechanisms involving JNK have recently been revealed, such as JNK/DLK positive feedback [34]. We predicted that c-Jun can bind to the promoter of CLDN6 gene. ChIP experiment further verified it (Fig. 6H). Thus, we speculated there may be a feedback mechanism involving CLDN6 and JNK/c-Jun pathway. We observed that c-Jun knockdown reduced CLDN6 expression (Fig. 6I; Supplementary Fig. 6B). Consistently, depletion of JNK also resulted in a significant reduction in CLDN6 expression (Fig. 6J). These data indicated that JNK/c-Jun pathway feedback regulated CLDN6 expression.

### The clinical correlation between CLDN6, WIP and LC3 expression in breast cancer patients

**Fig. 7:**
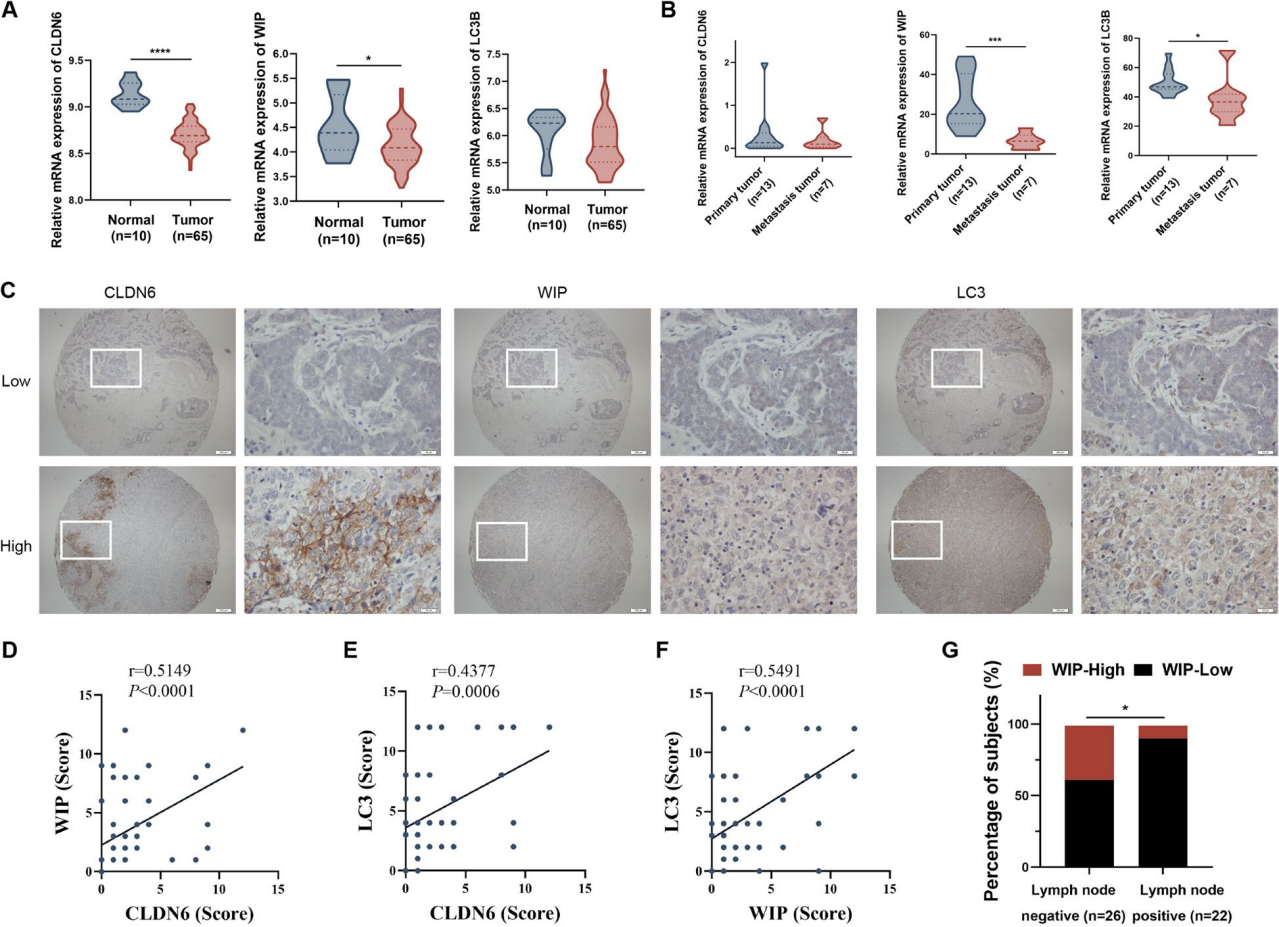
The clinical correlation between CLDN6, WIP and LC3 expression in breast cancer patients. **A** The mRNA expression of CLDN6, WIP and LC3 in adjacent normal and breast cancer tissues in the GEO database GSE103512. **B** The mRNA expression of CLDN6, WIP and LC3 in primary breast tumor tissues and metastatic tissues in the GEO database GSE191230. **C** Representative IHC images of low and high CLDN6, WIP and LC3 expression in breast cancer tissues. Scale bar, 200 µm (left), 20 µm (right). **D-F** Correlation analysis between CLDN6 and WIP (**D**), CLDN6 and LC3 (**E**), and WIP and LC3 (**F**) in breast cancer tissue microarray. Pearson correlation test, *n* = 58. **G** Percentage of high and low expression of WIP in breast cancer patients with lymph node-negative (*n* = 26) and node-positive (*n* = 22). *P < 0.05, ***P < 0.001, and ****P < 0.0001

To validate the relationship between CLDN6, WIP and LC3 expression and metastasis in breast cancer patients, we analyzed their mRNA expression with the GEO database. Data in GSE103512 showed that the level of CLDN6 and WIP in breast tumor tissues was lower than normal tissues (Fig. 7A). Data in GSE191230 showed that WIP and LC3 expression was lower in metastatic tissues compared with primary breast tumor tissues (Fig. 7B).

Next, we evaluated the expression of CLDN6, WIP and LC3 in tumor samples by IHC of the tissue microarray (Fig. 7C). Pearson correlation analyses indicated that CLDN6 was positively correlated with WIP and LC3, and that WIP was positively correlated with LC3 in breast cancer tissues (Fig. 7D-F). As shown in Table 1, WIP expression was significantly associated with lymph node metastasis. In the lymph node-positive group, the proportion of patients with low WIP expression was higher than the lymph node-negative group (Fig. 7G). In this study, there was no correlation between CLDN6 and LC3 expression and clinical parameters, which may be explained by the limited sample size.

**Table 1:** The association between CLDN6, WIP and LC3 expression and clinical characteristics of breast cancer patients

Clinicopathologic	CLDN6		*P*	WIP		*P*	LC3		*P*
**Features**	**Low**	**High**		**Low**	**High**		**Low**	**High**	
**Age**			0.8146			0.511			0.1763
≤ 57 years	21	5		21	5		20	6	
> 57 years	26	4		22	8		18	12	
**Tumor size**			0.7645			0.1839			0.5923
≤ 3 cm	30	6		25	11		24	12	
> 3 cm	16	3		17	2		14	5	
**Pathology grade**			0.1881			0.8305			0.5447
I—II	20	6		20	6		19	7	
II—III	27	2		23	6		19	10	
**Lymph node**			0.7808			0.0192*			0.3666
Negative	22	4		16	10		17	9	
Positive	17	5		20	2		17	5	
**TNM stage**			0.294			0.2963			0.3305
0—II	35	5		29	11		26	14	
III—IV	13	5		16	2		14	4	

^*^P < 0.05

## Discussion

As a tight junction protein, CLDN6 has emerging roles in epithelial tumors besides performing traditional barrier function. CLDN6 regulates the signaling effects mainly through the PBM at its carboxyl terminus, thereby affecting multiple biological behaviors of tumors [35]. In this study, we focused on the effect and mechanism of CLDN6 in inhibiting breast cancer metastasis.

Autophagy plays a fundamental role in cellular, tissue and organismal homeostasis [36]. There are limited reports on the effects of CLDNs-mediated autophagy. It was reported that CLDN5 knockout decreased autophagy in endothelial cells after hypoxia induction [37]. Wu et al. found that CLDN1 upregulation significantly increased autophagy flux in esophageal squamous carcinoma cells [38]. Our study demonstrated that CLDN6 overexpression induced autophagy. Recently, autophagy has been considered to inhibit tumor metastasis through multiple pathways [39]. Autophagy inhibits tumor metastasis by targeting ras homolog family member A or suppresses EMT, etc. However, depending on different tumor cell types and tumor microenvironment, autophagy can also promote tumor metastasis [15, 40]. In breast cancer, autophagy-deficient cells exhibited a higher capacity to form metastases by degrading Neighbor to BRCA1, the autophagy cargo receptor [41]. Consistently, we found that treatment with autophagy inhibitor reversed the inhibitory effect of CLDN6 overexpression on breast cancer metastasis both in vivo and vitro, which suggested that CLDN6 suppressed breast cancer metastasis via autophagy. Moreover, inhibition of autophagy rescued the inhibition of N-cadherin and vimentin induced by CLDN6 overexpression, indicating that CLDN6-mediated autophagy may degrade N-cadherin and vimentin to inhibit metastasis. Our findings illustrate the role of CLDN6-mediated autophagy in breast cancer metastasis, which makes CLDN6 possible as a biomarker to monitor autophagy status of breast cancer in vivo.

Autophagy is cross-regulated in cancer cells by many signaling pathways. These pathways regulate autophagy by influencing autophagy-related proteins [42, 43]. However, recent studies reported that actin cytoskeleton can regulate autophagy by directly affecting the autophagosome membrane [16, 17]. CLDNs are transmembrane proteins that can directly bind to proteins such as ZO-1, providing a link to the actin cytoskeleton for transducing regulatory signals to and from tight junctions [44]. CLDN1 affected the expression and activity of proteins that are associated with actin cytoskeleton remodeling [45]. Our study found that CLDN6 overexpression led to actin cytoskeleton rearrangement in breast cancer cells. Actin cytoskeleton was transferred from actin fibers at the cell edge to punctuated F-actin in the cytoplasm. Therefore, we wanted to explore the role of actin cytoskeleton in CLDN6-mediated autophagy. We found that p-Arp3 was co-localized with LC3, which indicated that actin cytoskeleton was localized in autophagosome. CK666 treatment attenuated the promotion of autophagic flux by CLDN6, suggesting that CLDN6 mediated autophagy through actin cytoskeleton. Our findings provide a new explanation for the regulation of CLDNs-mediated autophagy, through the actin cytoskeleton.

NPFs are key proteins that mediate actin cytoskeleton assembly [25]. It has been reported that NPFs are recruited to autophagosome to regulate different stages of autophagy [16]. JMY, a member of WHAMM Family, is recruited to autophagosome by binding to LC3 to promote autophagosome formation [18]. After autophagosome formation, WHAMM is recruited to autolysosomes membrane through PI(4,5)P_2_, to promote autolysosome reformation [21]. Moreover, deletion or down-regulation of WASP, WAVE and WASH blocks autophagy [20, 23, 46]. There are few studies on the relationship between CLDNs and NPFs. Astrid Escudero-Esparza et al. found that modulation of CLDN5 regulated N-WASP protein expression in MDA-MB-231 cells [47]. WIP was first identified as a partner of WASP, and regulates actin polymerization dependent on or independent of WASP [48]. In the present study, we found CLDN6 overexpression upregulated mRNA and protein expression of WIP. WIP knockdown resulted in actin cytoskeleton rearrangement and inhibited autophagy in CLDN6-overexpressing breast cancer cells. Moreover, actin cytoskeleton lost the effect of regulating autophagy after WIP knockdown. These indicated that WIP was critical for CLDN6 to regulate autophagy via the actin cytoskeleton. This is the first study to find that WIP regulates autophagy. Furthermore, our findings enriched the association between CLDNs, actin cytoskeleton and autophagy.

Aberrant expression of WIP contributed to metastasis of several malignancies [49]. Esther García found that WIP promoted migration and invasion of breast cancer cells by promoting the formation of invadopodia [50]. But we found different results. In our findings, upregulation of WIP by CLDN6 overexpression did not enhance pseudopodia at the cell edge. Moreover, WIP knockdown reversed the inhibitory effect of CLDN6 on breast cancer metastasis. To investigate the reasons for the difference, we found that WIP co-localized with LC3 and WIP interacted with LC3. Thus, upregulation of WIP by CLDN6 overexpression was recruited to autophagosome by LC3 to participate in autophagy, and cannot generate pseudopodia at the cell edge. Therefore, WIP inhibited breast cancer metastasis through autophagy, not through pseudopodia in CLDN6-overexpressing breast cancer cells.

The regulation of CLDN6 on WIP has not been reported. In this study, c-Jun was identified as a transcription factor of WIP regulated by CLDN6. The regulation of c-Jun is heavily dependent on post-translational modifications, and c-Jun is mainly phosphorylated by mitogen-activated protein kinases (MAPKs) [51]. JNK pathway is one of three well-characterized MAPKs pathways. The JNK-mediated phosphorylation of c-Jun enhances its binding to gene promoters, thus increasing its transcriptional efficiency [52]. In the present study, we found that CLDN6 overexpression upregulated the expression levels of JNK and p-JNK, as well as the expression of p–c-Jun. Similarly, Jong Min Baek et al. found that CLDN11 upregulated the expression of p-JNK in mouse osteoblasts [53]. Hong Liu et al. found that CLDN4 overexpression increased p-JNK protein expression in TE1 cells [54]. However, the interaction mechanism between CLDNs and the JNK pathway is still unclear. JNK-interacting protein 1 (JIP1) plays a role in JNK pathway activation by binding to JNK through its own JNK-binding domain (JBD) [55]. Jiyoung Moon et al. constructed a mutant JIP1 to disrupt JBD resulting in reduced binding affinity of JIP1 for JNK. Then they found that fusion of PDZ domain to mutant JIP1 can re-recruit JNK and restore JNK pathway activation [56]. Ethan L et al. found that E6 mutants lacking the carboxyl-terminal PBM failed to phosphorylate JNK [57]. These studies demonstrated that PDZ domains and PBM played important roles in JNK signaling. We created a mutant of CLDN6 (CLDN6ΔPBM) affecting its PBM and found that CLDN6ΔPBM failed to upregulate JNK protein expression and c-Jun activation. In CLDN6-overexpressing breast cancer cells, CLDN6 interacted with JNK, but CLDN6ΔPBM lost this function. Thus, CLDN6 interacted with JNK by binding to PDZ-domain-containing proteins via its PBM, which is required for JNK/c-Jun activation. But the details of their interaction and how this binding leads to elevated JNK expression need to be further investigated.

There is a positive feedback regulation mechanism in the JNK pathway such as MLK3 and DLK, which can directly be phosphorylated by activated JNK, exerting positive feedback [34, 58]. Stem cell factor activated JNK/c-Jun, and c-Jun bound to CLDN3 promoter region to promote CLDN3 expression in HT-29 cells [59]. Similarly, we found that JNK and c-Jun knockdown resulted in down-regulation of CLDN6 mRNA and protein expression. The promoter region of CLDN6 was predicted to have potential binding sites for c-Jun, and further verification by ChIP experiments was performed. These results suggested that c-Jun transcriptionally regulated CLDN6. Our study is the first to uncover a positive feedback loop of CLDN6 and JNK/c-Jun signaling, which may increase the inhibitory effect of CLDN6 on breast cancer metastasis.

## Conclusions

**Fig. 8:**
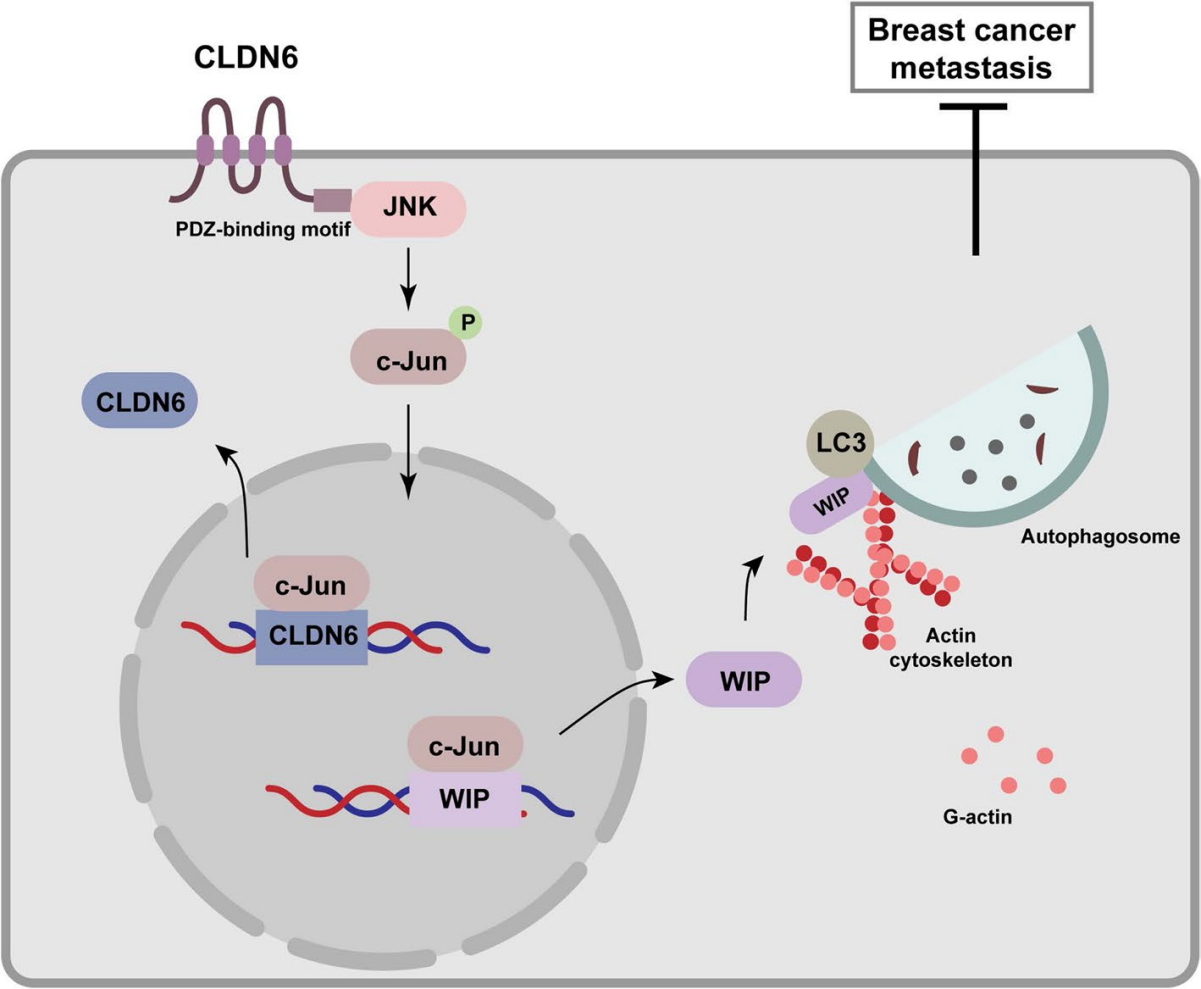
A proposed model for the regulatory mechanism of CLDN6-mediated autophagy inhibiting breast cancer metastasis

In summary, we found that CLDN6 inhibited breast cancer metastasis via autophagy in vitro and vivo. Mechanistically, CLDN6 interacted with JNK via PBM to upregulate JNK protein expression and activate JNK/c-Jun pathway, thereby enhancing the transcriptional activity of c-Jun. On the one hand, c-Jun upregulated CLDN6 expression, forming a positive feedback loop. On the other hand, c-Jun bound to the gene promoter region of WIP to upregulate WIP expression. WIP was recruited by LC3 and promoted actin cytoskeleton assembly, ultimately participating in autophagy and inhibiting cancer metastasis (Fig. 8). Given the heterogeneity of breast cancer and the fact that cancer treatment may require long-term administration of certain autophagy modulators [60], CLDN6 may act as a biomarker to monitor the autophagy status in vivo. Our study enriches the theoretical basis for CLDN6 as a potential biomarker for breast cancer diagnosis and therapy.

## Supplementary Information

**Additional file 1: Supplementary Fig. 1.** Identification of CLDN6 overexpression in MCF-7 and MDA-MB-231 cells. **Supplementary Fig. 2.** CLDN6-mediated autophagy regulates EMT. **Supplementary Fig. 3.** CLDN6 increases WIP expression. **Supplementary Fig. 4.** ImageJ was used for colocation analysis of LC3 and WIP. **Supplementary Fig. 5.** The Correlation between JNK/c-Jun and WIP mRNA. **Supplementary Fig. 6.** C-Jun regulates the expression of WIP and CLDN6 at Transcriptional Level. **Supplementary Table 1.** Antibodies utilized in this study. **Supplementary Table 2.** Primers utilized in this study.

## Data Availability

The datasets used and/or analysed during the current study are available from the corresponding author on reasonable request.

## Abbreviations

PBM: PDZ-binding motif
NPF: Nucleation-promoting factors
WIP: WASP-interacting protein
DMEM: Dulbecco’s modified Eagle’s medium
FBS: Fetal bovine serum
IF: Immunofluorescent
shRNA: Short hairpin RNA
TEM: Transmission electron microscopy
ChIP: Chromatin immunoprecipitation
Co-IP: Co-immunoprecipitation
CQ: Chloroquine
H&E: Hematoxylin and eosin
IHC: Immunohistochemistry
SD: Standard deviation
MAPKs: Mitogen-activated protein kinases
JIP1: JNK-interacting protein 1
JBD: JNK-binding domain
