# Supplementary material for: CLDN6 inhibits breast cancer metastasis through WIP-dependent actin cytoskeleton-mediated autophagy
Source: J Exp Clin Cancer Res. 2023 Mar 20;42:68. doi: 10.1186/s13046-023-02644-x (PMC10026481; doi:10.1186/s13046-023-02644-x)
Supplement: Supplementary file 1 — Additional file 1: Supplementary Fig. 1. Identification of CLDN6 overexpression in MCF-7 and MDA-MB-231 cells. Supplementary Fig. 2. CLDN6-mediated autophagy regulates EMT. Supplementary Fig. 3. CLDN6 increases WIP expression. Supplementary Fig. 4. ImageJ was used for colocation analysis of LC3 and WIP. Supplementary Fig. 5. The Correlation between JNK/c-Jun and WIP mRNA. Supplementary Fig. 6. C-Jun regulates the expression of WIP and CLDN6 at Transcriptional Level. Supplementary Table 1. Antibodies utilized in this study. Supplementary Table 2. Primers utilized in this study. [file 13046_2023_2644_MOESM1_ESM.docx]

**Supplementary Materials**

# CLDN6 inhibits breast cancer metastasis through WIP-dependent actin cytoskeleton-mediated autophagy

Yuan Dong^1^, Qiu Jin^1^, Minghao Sun^1^, Da Qi^1^, Huinan Qu^1^, Xinqi Wang^1^ and Chengshi Quan^1,^ *

* Corresponding Author

^1^ The Key Laboratory of Pathobiology, Ministry of Education, College of Basic Medical Sciences, Jilin University, 126 Xinmin Avenue, Changchun, 130021, Jilin, China.

E-mails: [quancs@jlu.edu.cn](mailto:quancs@jlu.edu.cn)

**Supplementary Fig. 1**


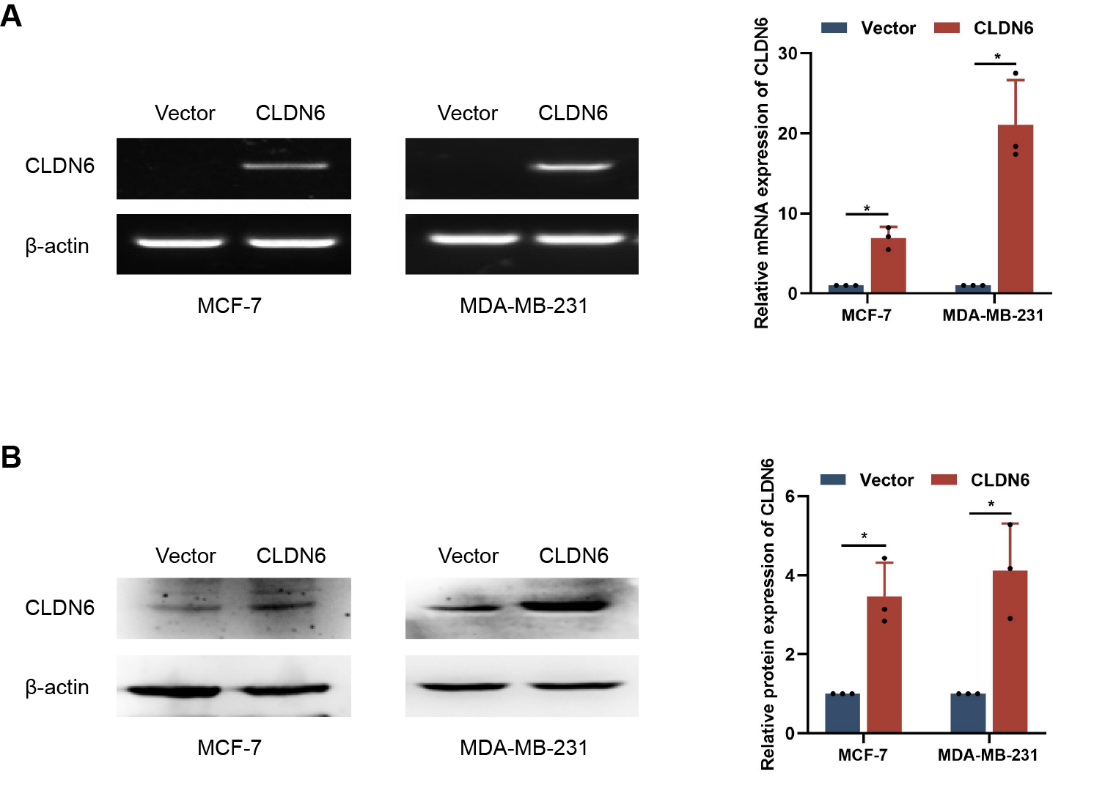


**Supplementary Fig. 1** Identification of CLDN6 overexpression in MCF-7 and MDA-MB-231 cells. **(A-B)** RT-PCR **(A)** and Western blot **(B)** validation of CLDN6 overexpression in MCF-7 and MDA-MB-231 cells. Results were from three independent experiments. **P* < 0.05.

**Supplementary Fig. 2**

**
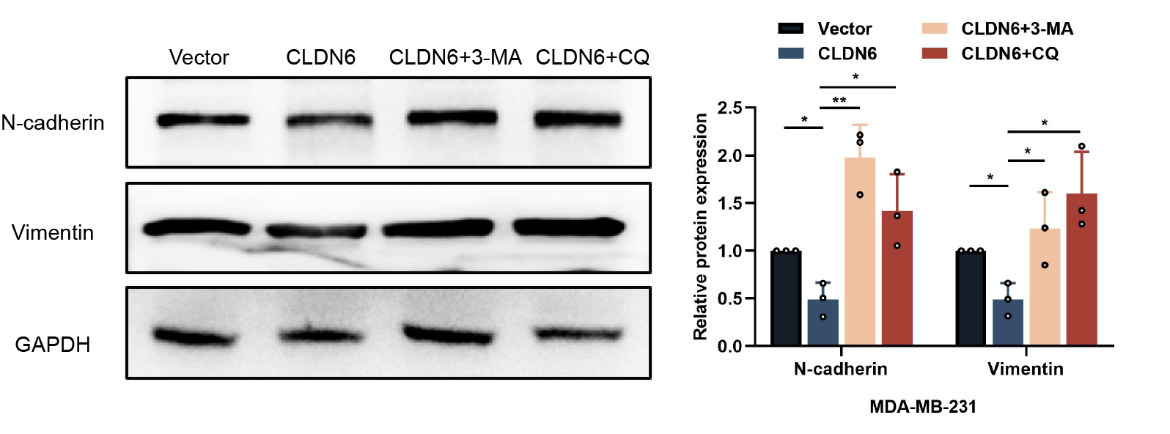
**

**Supplementary Fig. 2** CLDN6-mediated autophagy regulates EMT. Western blot showed levels of N-cadherin and vimentin after 3-MA and CQ treatment in MDA-MB-231/CLDN6 cells. **P* < 0.05, ***P* < 0.01.

**Supplementary Fig. 3**


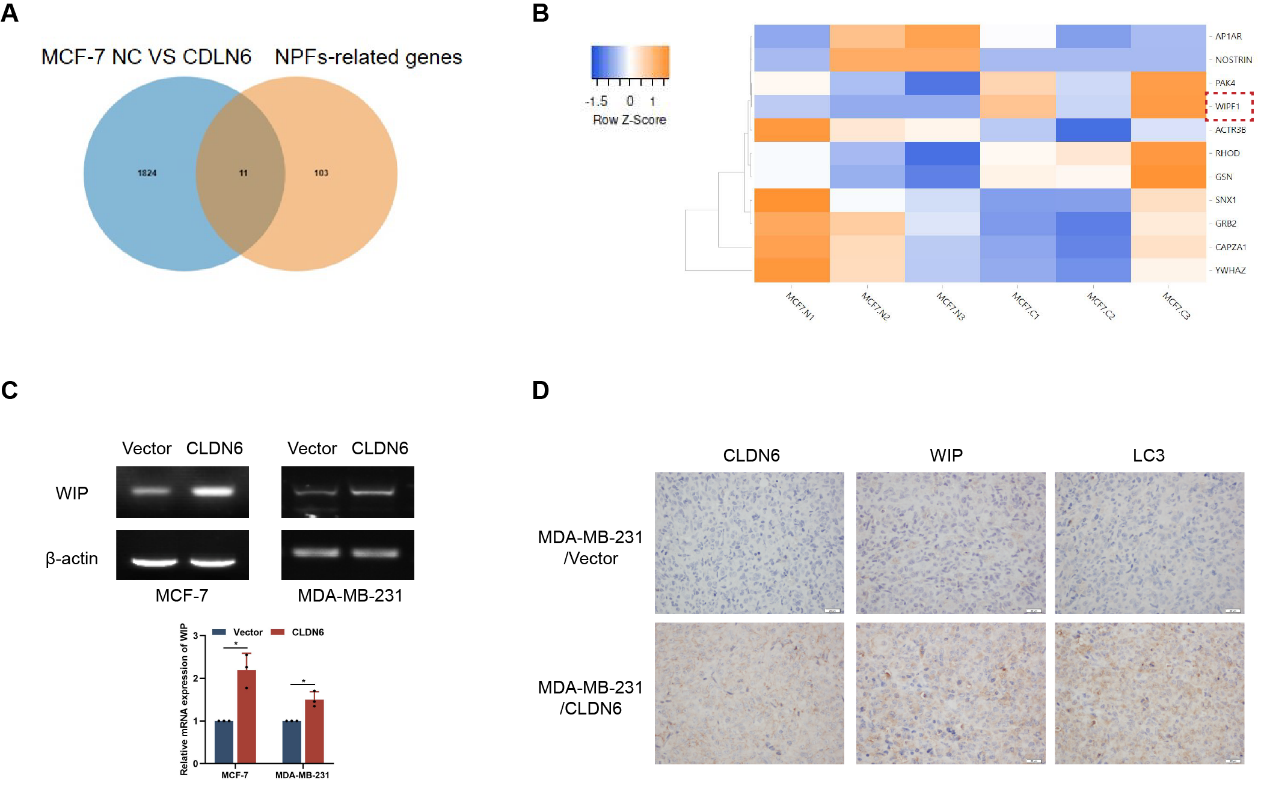


**Supplementary Fig. 3** CLDN6 increases WIP expression. **(A)** Overlap of changed genes affected by CLDN6 overexpression and NPFs-related genes. **(B)** Heatmap showed the subset of 11 genes identified. **(C)** RT-PCR showed levels of WIP mRNA in MCF-7 and MDA-MB-231 cells with or without CLDN6 overexpression. Results were from three independent experiments. **(D)** Representative IHC images of CLDN6, WIP and LC3 in the lung metastatic tumor tissues. Scale bar: 20 μm. **P* < 0.05.

**Supplementary Fig. 4**


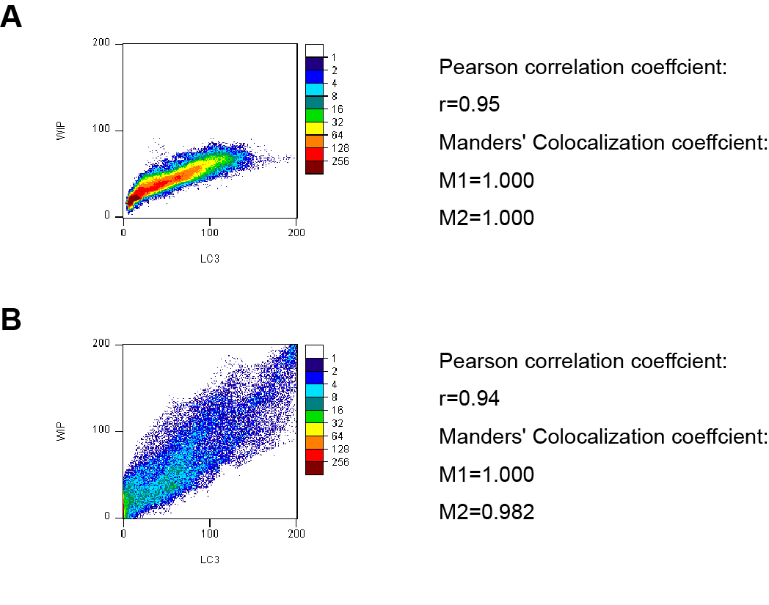


**Supplementary Fig. 4** ImageJ was used for colocation analysis of LC3 and WIP. **(A-B)** Pearson correlation analysis in MCF-7/CLDN6 **(A)** and MDA-MB-231/CLDN6 **(B)** showed that r > 0.5. Manders^,^ colocation coefficient showed that M1 > 0.5 and M2 > 0.5.

**Supplementary Fig. 5**


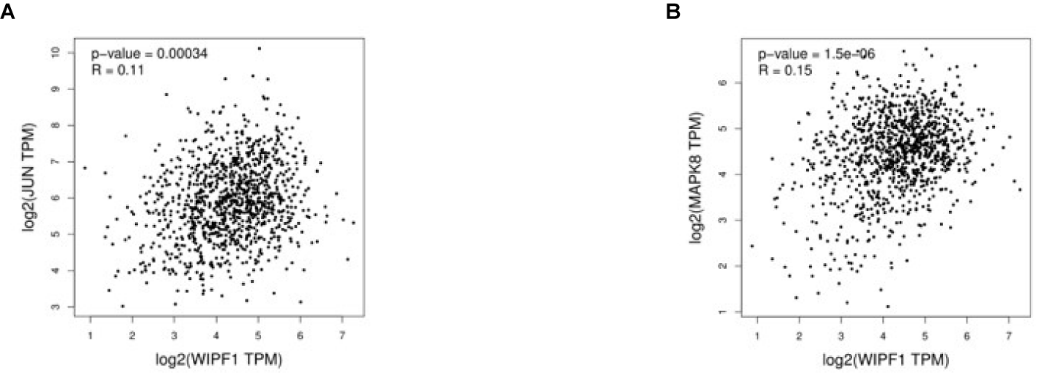


**Supplementary Fig. 5** The Correlation between JNK/c-Jun and WIP mRNA. **(A-B)** Data from the TCGA demonstrated that WIP expression was positively correlated with c-Jun expression **(A)** as well as JNK expression **(B)**.

**Supplementary Fig. 6**


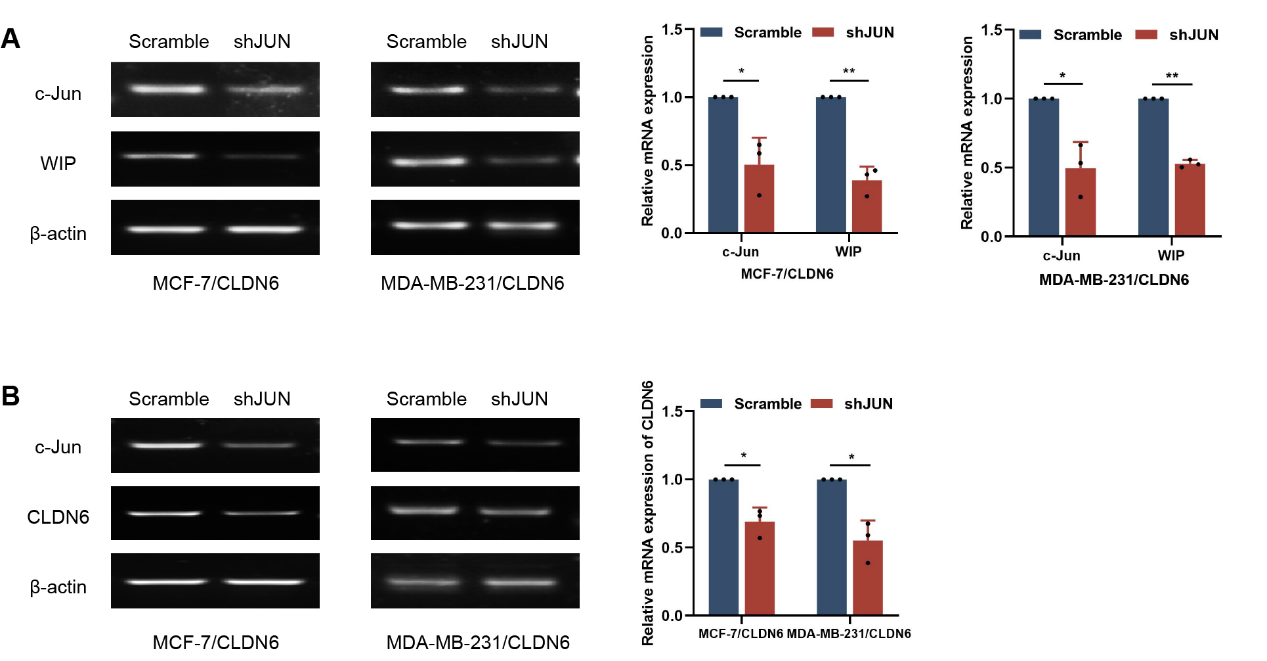


**Supplementary Fig. 6** C-Jun regulates the expression of WIP and CLDN6 at Transcriptional Level. **(A)** RT-PCR showed mRNA levels of WIP in MCF-7/CLDN6 and MDA-MB-231/CLDN6 cells with or without c-Jun knockdown.

**(B)** RT-PCR showed mRNA levels of CLDN6 in MCF-7/CLDN6 and MDA-MB-231/CLDN6 cells with or without c-Jun knockdown. **P* < 0.05, ***P* < 0.01.

**Supplementary Table 1 Antibodies utilized in this study.**

| **Antibody** | **Application** | **Source** | **Catalog number** |
| --- | --- | --- | --- |
| CLDN6  CLDN6 | WB/IHC/IP/IF  IP | Santa Cruz  Cell Signaling Technology | sc-393671  62831S |
| LC3  LC3B | WB/IHC/IP/IF  IF | Proteintech  Abclonal | 14600-1-AP  A17424 |
| p62 | WB | Proteintech | 18420-1-AP |
| ATG5 | WB | Cell Signaling Technology | 12994T |
| ATG7 | WB | Cell Signaling Technology | 8558T |
| β-actin  N-cadherin  Vimentin  p-Arp3 | WB  WB  WB  IF | Proteintech  Proteintech  Proteintech  Bioss | 66009-1-Ig  22018-1-AP  10366-1-AP  Bs-8999R |
| WIP | WB/IHC/IP/IF | Santa Cruz | sc-271113 |
| c-Jun | WB/ChIP | Cell Signaling Technology | 9165S |
| p-c-Jun | WB | Cell Signaling Technology | 3270T |
| JNK | WB/IP | Santa Cruz | sc-137018 |
| JNK | IF | Proteintech | 24164-1-AP |
| p-JNK | WB | Cell Signaling Technology | 4668T |
| GAPDH | WB | Proteintech | 60004-1-Ig |

**Supplementary Table 2 Primers utilized in this study.**

| **For RT-PCR** |  |
| --- | --- |
| CLDN6  WIP  β-actin | CTGCCCATGTGGAAGGTGAC  GGTAGACCAGCAAGCCGAAC  AGCCTCAGAGGAACCGAATG  CGGACTTGATTGAATGGGTCTTG  CAGAGCCTCGCCTTTGCCGATCC |
|  | CCTTGCACATGCCGGAGCCGT |
| c-Jun | CTGCGTCTTAGGCTTCTCC |
|  | CTCGCCCAAGTTCAACAA |
| **For ChIP-PCR** |  |
| WIP | CACTGGCAGCAAAACGACTC |
| CLDN6 | CAAGGTCCACACGCATCTGA  CATAAATTAGCTGGGCACGGTGG  ACGTCCTAAGGTACAAATTAAAGGGCATAC |
